# Spin texture induced by non-magnetic doping and spin dynamics in 2D triangular lattice antiferromagnet *h*-Y(Mn,Al)O_3_

**DOI:** 10.1038/s41467-021-22569-3

**Published:** 2021-04-16

**Authors:** Pyeongjae Park, Kisoo Park, Joosung Oh, Ki Hoon Lee, Jonathan C. Leiner, Hasung Sim, Taehun Kim, Jaehong Jeong, Kirrily C. Rule, Kazuya Kamazawa, Kazuki Iida, T. G. Perring, Hyungje Woo, S.-W. Cheong, M. E. Zhitomirsky, A. L. Chernyshev, Je-Geun Park

**Affiliations:** 1 Center for Quantum Materials, Seoul National University, Seoul, 08826 Republic of Korea; 2 Center for Correlated Electron Systems, Institute for Basic Science, Seoul National University, Seoul, 08826 Republic of Korea; 3 Department of Physics and Astronomy & Institute of Applied Physics, Seoul National University, Seoul, 08826 Republic of Korea; 4 Center for Theoretical Physics of Complex Systems, Institute for Basic Science, Daejeon, 34126 Republic of Korea; 5 Department of Physics, Incheon National University, Incheon, 22012 Republic of Korea; 6 Physik-Department, Technische Universität München, D-85748 Garching, Germany; 7 Australian Nuclear Science and Technology Organisation, Lucas Heights, 2234 NSW Australia; 8 Comprehensive Research Organization for Science and Society (CROSS), Tokai, Ibaraki, 319-1106 Japan; 9 ISIS Pulsed Neutron and Muon Source, STFC Rutherford Appleton Laboratory, Didcot, Oxfordshire OX11 0QX United Kingdom; 10 Department of Physics, Brookhaven National Laboratory, Upton, NY 11973 USA; 11 Rutgers Center for Emergent Materials and Department of Physics and Astronomy, Rutgers University, Piscataway, New Jersey 08854 USA; 12 Université Grenoble Alpes, CEA, IRIG, PHELIQS, 38000 Grenoble, France; 13 Department of Physics and Astronomy, University of California, Irvine, CA 92697 USA

**Keywords:** Structure of solids and liquids, Magnetic properties and materials

## Abstract

Novel effects induced by nonmagnetic impurities in frustrated magnets and quantum spin liquid represent a highly nontrivial and interesting problem. A theoretical proposal of extended modulated spin structures induced by doping of such magnets, distinct from the well-known skyrmions has attracted significant interest. Here, we demonstrate that nonmagnetic impurities can produce such extended spin structures in *h*-YMnO_3_, a triangular antiferromagnet with noncollinear magnetic order. Using inelastic neutron scattering (INS), we measured the full dynamical structure factor in Al-doped *h*-YMnO_3_ and confirmed the presence of magnon damping with a clear momentum dependence. Our theoretical calculations can reproduce the key features of the INS data, supporting the formation of the proposed spin textures. As such, our study provides the first experimental confirmation of the impurity-induced spin textures. It offers new insights and understanding of the impurity effects in a broad class of noncollinear magnetic systems.

## Abstract (web summary)

In geometrically frustrated magnets small perturbations, such as non-magnetic impurities, can significantly alter the ground state. Here, using inelastic neutron scattering, Park et al. explore such a situation in Al-doped h-YMnO3, where the Aluminum dopants induce an extended spin structure.

## Introduction

In many geometrically frustrated magnets, a weak perturbation can potentially induce new competing ground states, leading to an extremely rich phase diagram. A particularly important but less explored case is that of the frustrated system with defects, i.e., nonmagnetic impurities, which are ubiquitous in real-world materials. Combined with the geometrical frustration, impurities can introduce several nontrivial effects in a wide variety of frustrated magnets. For example, extensive search for a genuine quantum spin liquid (QSL) state in frustrated magnets has been consistently plagued by the impurity issues, e.g., herbertsmithite and YbMgGaO_4_ as the *S* = 1/2 kagome^1–3^ and triangular^4–6^ QSL candidates, respectively. It has been theoretically pointed out that impurities themselves can induce continuum-like excitations in geometrically frustrated magnets, which is difficult to distinguish from the genuine signature of a QSL state. Other examples are a rich phase diagram due to impurities in a triangular lattice antiferromagnet^7^ (TLAF), an impurity-induced spin-glass state in the frustrated spinel^8–10^ and spin ice^11,12^ systems, suppression of the QSL state due to impurities^13^, and a disorder-induced classical^14,15^ and quantum^16^ QSL state, to name a few.

**Fig. 1:**
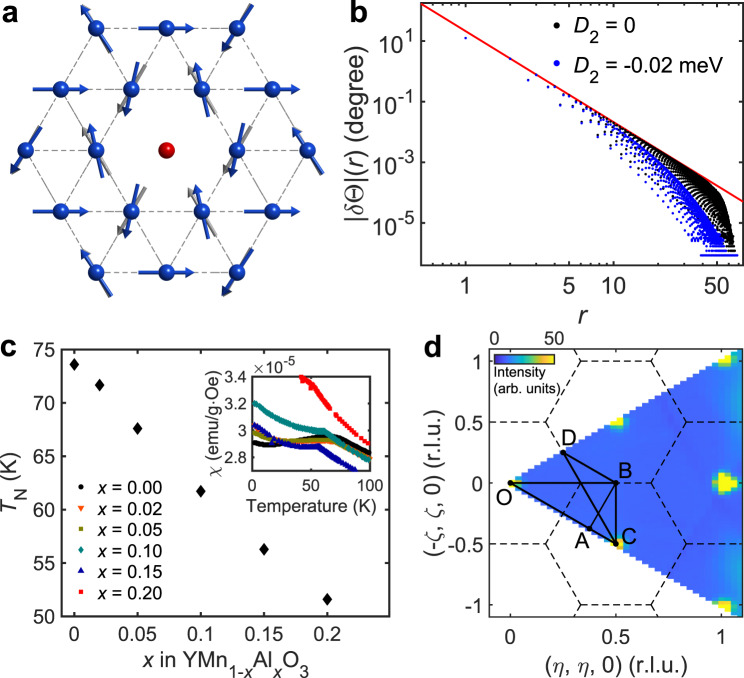
Formation of a spin texture in 2D-TLAF *h-*Y(Mn,Al)O_3_. **a** Local spin canting induced by a vacancy in a triangular lattice antiferromagnet (blue arrows) and original 120^*◦*^ magnetic order (gray arrows). **b** Distance dependence of the canting angle $|{\rm{\delta }}\Theta (r)|$ in the spin texture around a vacancy determined from the numerical simulations. Black (Blue) dots denote the simulation with *D*_2_ = 0 (−0.02) meV, repectively. The red line is a guided plot of the 1/*r*^3^ behavior. **c** Doping dependence of the transition temperature *T*_N_. The inset denotes the magnetic susceptibility of YMn_1-*x*_Al_*x*_O_3_ as a function of temperature, from which the transition temperatures (*T*_N_) were determined. **d** Two-dimensional reciprocal space of YMnO_3_ with the labels of high-symmetric points and the high-symmetric lines used in Fig. 2. Color plot shows magnetic Bragg peaks of *h-*YMn_0.85_Al_0.15_O_3_ measured by neutron scattering.

One of the salient features resulting from a nontrivial interplay between the geometrical frustration and disorder is a significant modification of spins’ directions near impurities. In frustrated magnets, a vacancy gives rise to a partial relief of local frustration around it, which leads to a sizable reorientation of spins proximate to the impurity site (Fig. 1a). In a coplanar magnetic ground state with a well-defined easy plane, the reorientation only consists of in-plane components. The reorientation at the six nearest sites points toward the opposite direction to the effective impurity moment, leading to a screening of the effective impurity moment. Subsequently, spins at the next-nearest sites are affected by the nearest sites’ reorientation, leading again to the canting toward the direction opposite to the former reorientation (See Fig. S1 for demonstration of the spatial structure of the spin reorientation). Such a spatially-correlated pattern of the spin reorientation acts as a partial screening of the effective impurity moments, resulting in an uncompensated fractional impurity moment^17^, among other phenomena.

Moreover, the spins’ canting angle decays algebraically to the distance from the vacancy^17–19^ (Fig. 1b), resulting in a formation of an extended spin object around the vacancy called *spin texture*^17,20,21^. This impurity-induced texture can affect the spin dynamics and the ground state of the original system, which determines the thermal and spin transport properties of the material. Therefore, it may have a broad applicability to several fields in magnetism, not to mention its importance as a new way of generating large spin objects analogous to a skyrmion. However, despite such outstanding interests, there has been no experimental demonstration of the impurity-induced spin textures as of yet.

*h*-YMn_1-*x*_Al_*x*_O_3_ is a unique model system to study dilution effects in a frustrated magnet, where non-magnetic Al^3+^ ions are doped into the Mn^3+^ triangular lattice^22,23^. Pure *h*-YMnO_3_ has noncollinear 120° magnetic order below *T*_N_ = 74 K due to the geometrical frustration. The spin dynamics of *h*-YMnO_3_ have been well established by the previous studies^24–31^, which used a simple model Hamiltonian (Eq. 1) with some additional terms from magnon-magnon/phonon coupling. Most importantly, increasing Al concentration *x* up to 0.2 does not change the crystalline symmetry but only reduces the antiferromagnetic transition temperature (Fig. 1c) and the magnetic moments of Mn^3+^ gradually, enabling a systematic study of the dilution effect on the magnetism of a triangular lattice antiferromagnet (TLAF)^23^. While a large single crystal of *h*-YMn_1-*x*_Al_*x*_O_3_ suited for inelastic neutron scattering (INS) is available^23^, previous studies on this material focused only on the crystal structure and its bulk properties^23,32,33^.

In this work, we report the full spin dynamics of *h*-YMn_1-*x*_Al_*x*_O_3_ studied by INS and model calculations. Our INS data of *h*-YMn_1-*x*_Al_*x*_O_3_ reveal the presence of magnon damping with clear momentum dependence, in accordance with the theoretical calculation incorporating the spin texture. Our result provides the first experimental evidence of the impurity-induced spin texture, and prompt a fundamentally new understanding of the impurity effects in a wide variety of non-collinear magnetic systems.

## Results and discussion

**Fig. 2:**
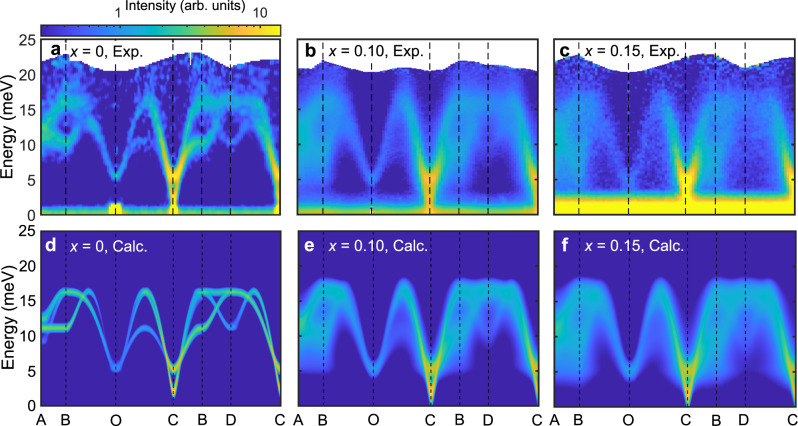
Magnetic excitation spectra of *h*-YMn_1-*x*_Al_*x*_O_3_. **a**–**c** INS spectra of **a** *h-*YMnO_3_ (ISIS), **b** *h-*YMn_0.9_Al_0.1_O_3_ (J-PARC) and **c** *h-*YMn_0.85_Al_0.15_O_3_ (J-PARC) measured at 5 K with the incident neutron energy of *E*_*i*_ = 30 meV. The scattering intensity of the data was integrated over the *c*^∗^-axis. **d**–**f** Theoretical INS cross-section of **d** *h-*YMnO_3_, **e** *h-*YMn_0.9_Al_0.1_O_3_, and **f** *h-*YMn_0.85_Al_0.15_O_3_, which include the instrumental resolution convolution as well as the data integration effect over the *c*^∗^-axis (see Methods).

**Fig. 3:**
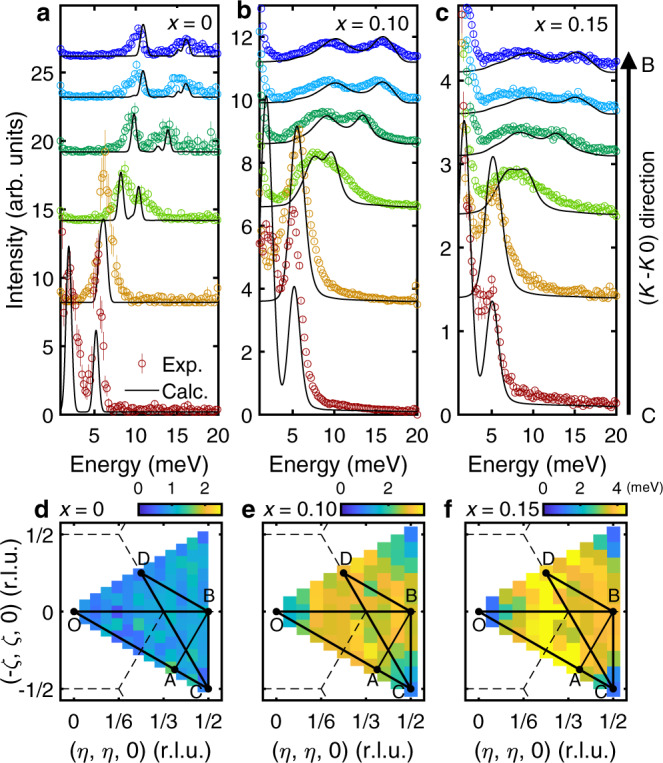
*Q-*dependent magnon linewidth broadening due to doping. **a**–**c** Constant ***Q***-cuts (colored circles) and theoretically calculated INS cross-sections (solid black lines) of **a** *h*-YMnO_3_, **b** *h*-YMn_0.9_Al_0.1_O_3_, and **c** *h*-YMn_0.85_Al_0.15_O_3_ at various **Q** points along the [*K*, -*K*, 0] direction (the C-B direction). Both the experimental data and the theoretical calculation results are rendered with the intensity integration along the *c*^∗^-axis. **d**–**f** Fitted intrinsic HWHM (Γ(***Q***)) of the magnon modes in **d** *h*-YMnO_3_, **e** *h*-YMn_0.9_Al_0.1_O_3_, and **f** *h*-YMn_0.85_Al_0.15_O_3_ over the full Brillouin zone, where the instrumental resolution effects are excluded (see Methods).

Figure 2a–c show the energy-momentum (**Q**) slices from the INS data of 0%, 10%, and 15% Al-doped *h*-YMnO_3_ single crystals along the high-symmetric lines (see Fig. 1d), which demonstrate the influence of the Al doping. In contrast to the clear INS spectra of the pristine *h*-YMnO_3_, the INS spectra of the Al-doped samples show broad magnon signals as expected for magnetic systems with impurities. Interestingly, however, the observed energy linewidth broadening is not uniform over the Brillouin zone but **Q**-dependent. A stack of constant **Q**-cuts along the B − C ([K − K 0] direction) in Fig. 3a–c supports this statement: near the magnetic zone center (C point), the linewidth of the magnon peaks is hardly changed with doping, whereas the magnon peaks near the zone boundary (B point) undergo a drastic broadening. For a more transparent demonstration of the **Q***-*dependent energy linewidth broadening, see Supplementary Fig. S5. For a deeper understanding of the **Q***-*dependent linewidth broadening, we analyzed the half-width at half-maximum (HWHM) of the magnon peaks (see Methods) over the full Brillouin zone (Fig. 3d–f), which was averaged for all magnon modes at each **Q**-point. We note that there is no clear **Q**-dependence of the HWHM in the pure *h*-YMnO_3_ (Fig. 3d). In *h*-Y(Mn,Al)O_3_, however, the HWHM steadily increases when the magnon branches get closer to the zone boundary, and reaches its maximum near the B point (equivalent to the M point in the reciprocal space of a triangular lattice). Another noticeable feature is the **Q***-*dependent magnon energy renormalization, manifested in the increasingly downward shift of the magnon dispersion along the A-B direction (Fig. 2a–c). Such **Q**-dependent energy linewidth broadening and renormalization of magnons may imply the presence of something beyond a simple dilution effect, as a point-like scattering potential made by an impurity is known to induce a **Q**-independent behavior of magnons generally^21,34,35^.

To explain the observed features, we have modeled the spin Hamiltonian of a diluted triangular lattice with randomly distributed vacancies (see Methods for details). We assumed that Al doping does not change the parameters of the spin Hamiltonian, which is plausible considering the simple linear relation between *x* and *T*_N_ (Fig. 1c)^36^. The spin dynamics of pure *h*-YMnO_3_ can be described by the following spin Hamiltonian suggested by the previous study:^24^

1 $${H}_{spin}={J}_{1}\mathop{\sum}\limits_{\langle ij\rangle }{{\boldsymbol{S}}}_{i}\cdot {{\boldsymbol{S}}}_{j}+{D}_{1}\mathop{\sum}\limits_{i}{({s}_{i}^{z})}^{2}+{D}_{2}\mathop{\sum}\limits_{i}{({{\boldsymbol{S}}}_{i}\cdot \hat{n})}^{2},$$

where *J*_1_ denotes the coupling constant of nearest-neighbor super-exchange interaction, and *D*_1_ and *D*_2_ are the size of the easy-plane anisotropy and the local easy-axis anisotropy along the direction of each spin ($\hat{n}$) in the 120˚ magnetic structure, respectively. Note that we assumed an ideal triangular lattice without trimerization present in *h*-YMnO_3_ to focus on the impurity effects for this work, as the change due to the trimerization would be marginal from the viewpoint of its spin dynamics. For model calculations with the trimerization effect, see the Supplementary Information (Fig. S2). We adopted the parameters from ref. ^24^ *J*_1_ = 2.5 meV, *D*_1_ = 0.28 meV, and *D*_2_ = − 0.02 meV. Note that both *D*_1_ and *D*_2_ contribute to forming the two gaps at 5 and 2 meV, respectively, leading to the double peak structure at the C point in both pure and Al-doped YMnO_3_ (Fig. 2).

First, we tested the ground state modification by a single impurity (see Methods) and compared it with our previous theoretical work results. Figure 1b shows the canting angle of spins $|{\rm{\delta }}\Theta (r)|$ as a function of the distance from the impurity site (*r)* on a logarithmic scale, both with and without the easy-axis anisotropy *D*_2_. For the directions of the spin canting, see Supplementary Fig. S1. Without the easy-axis anisotropy (*D*_2_=0), the canting angle follows asymptotically the algebraic decay law, 1/*r*^3^, indicated by the guide line^17^. The long-range spin texture is formed with the canting angle depending on the distance *r* as well as on the sublattice number^7^. At large distances (*r* > 30), the numerical data are affected by finite-size effects. Turning on *D*_2_, the canting angle decreases faster with distance, as expected for anisotropic models, but the spin texture remains almost intact at intermediate distances.

Using the relaxed spin configuration, including the spin texture and Eq. 1, we calculated the INS cross-section of YMn_1-*x*_Al_*x*_O_3_ at the level of linear spin-wave theory (LSWT); see Methods for the details. Figures 2d–f and 3a–c show the results of the calculations convoluted with the instrumental resolution for *x*=0, 0.1, and 0.15, which are in excellent agreement with our INS data (for the results without the resolution convolution, see Supplementary Figs. S7 and S8). These results suggest that despite the simplicity of LSWT, our model calculation has successfully captured the unusual features observed in the data: the **Q**-dependent energy linewidth broadening and the increase of the downward shift of the magnon dispersion along the A-B direction. Note that the discrepancy between the magnon dispersion along A-B in Figs. 2a and 2d (*x*=0) is due to the effect of magnon-phonon coupling present in *h*-YMnO_3_ (ref. ^24^), which was not considered in our model calculation.

Such a **Q**-dependent magnon lifetime in a diluted noncollinear magnet has already been suggested by a previous theoretical study^21^, which argued that the spin texture gives rise to **Q**-dependent magnon scattering. According to ref. ^21^, the impurity-induced spin texture generates a spatially-dispersed effective magnetic field around an impurity (unlike a point-like potential made by the impurity itself), which acts as a potential for the magnon Umklapp scattering. Importantly, this scattering makes the scattering rate strongly **Q**-dependent as exactly found in our experiments. As the effect mentioned above is already embedded in our model calculations, the **Q**-dependent behavior observed in both the data and the calculations would be from the magnons’ scattering on the spin textures. Note that similar **Q**-dependent magnon scattering was also suggested in the system with skyrmions, where the effective magnetic field from its topological texture acts as a scattering potential^37^. To verify that the observed behavior is unique characteristics of a frustrated magnet, we contrast it with a diluted non-frustrated square-lattice antiferromagnet’s spin dynamics. As shown in Supplementary Fig. S9, impurities in a square lattice create a flat localized mode, consistent with the previous INS study on a perovskite fluoride K(Co,Mn)F_3_ (ref. ^38^). However, one does not see any noticeable **Q**-dependent energy linewidth broadening or renormalization when increasing doping. Such distinctive difference comes from the absence of geometrical frustration (and, therefore, the absence of spin textures) in the square lattice antiferromagnet as opposed to the TLAF. Further theoretical analysis is needed for a comprehensive understanding of the specifics of the observed **Q**-dependence, such as why the most drastic changes in the spectrum due to dilution occur in the vicinity of the B point, which we leave for further studies.

**Fig. 4:**
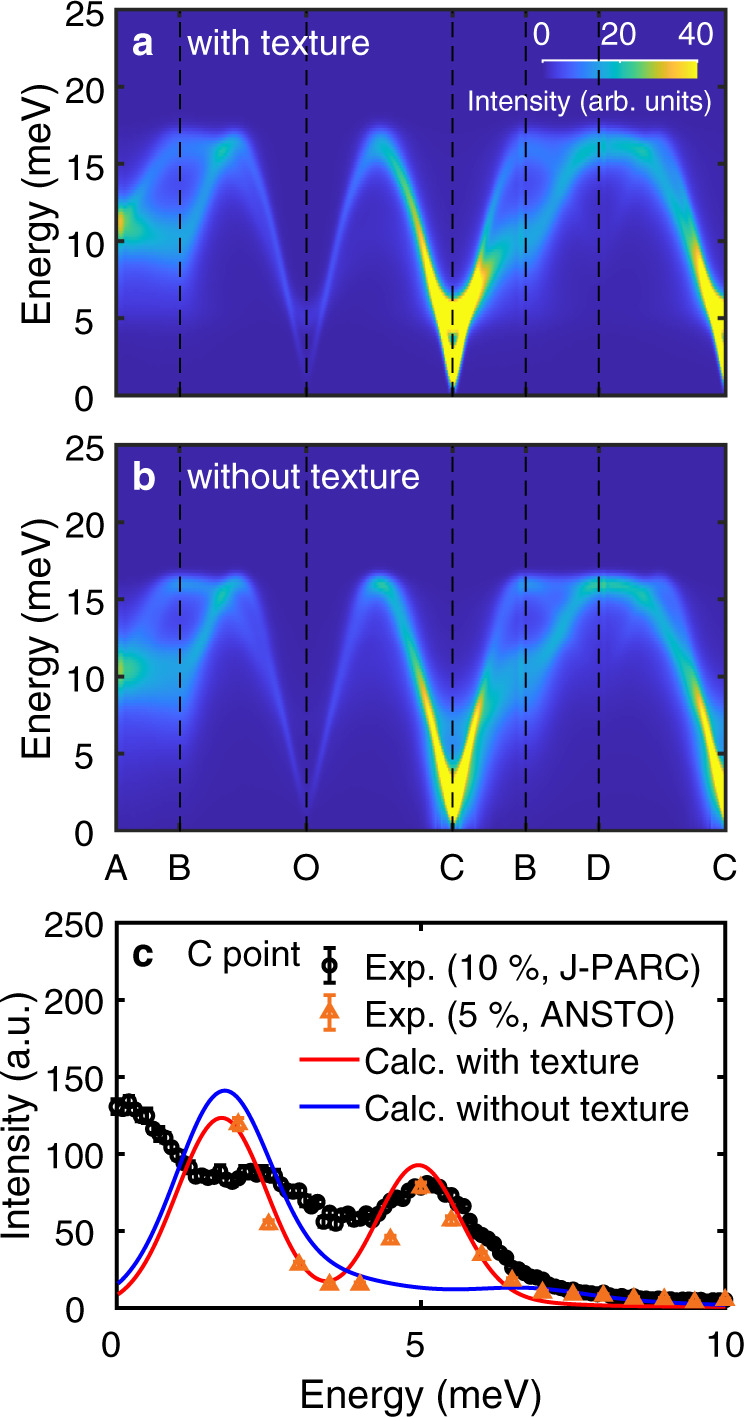
A role of spin textures in the spin dynamics of *h-*YMn_1-x_Al_x_O_3_. Theoretical spin-wave spectra of *h*-YMn_0.9_Al_0.1_O_3_ **a** with vacancies and spin textures, and **b** with vacancies but without spin textures. **c** A constant **Q**-cut at the C point, which demonstrates the suppression of the 5 meV mode in the calculation results without spin texture. Black and orange points are the INS data of *h*-YMn_0.9_Al_0.1_O_3_ and *h*-YMn_0.95_Al_0.05_O_3_, respectively. Blue and Red solid lines are the constant-**Q** cuts of **a** and **b**. Note that this figure’s calculation results do not include the effect of data integration along the *c*^∗^-axis.

To explicitly examine the spin textures’ role in the spin dynamics of *h*-Y(Mn,Al)O_3_, we have also calculated the INS cross-sections of a diluted triangular lattice without the feedback of the vacancy, i.e., without the spin texture. To perform such calculation, we artificially forced the spins to retain the 120˚ magnetic order for the calculation, which is similar to the approach used in ref. ^21^. It amounts to neglecting the feedback of the impurity onto the host spins, so that no texture is created. This allows us to separate scattering effects of the conventional dilution from the effects associated with extended textures. Figure 4a, b show the calculated magnon spectra of 10% Al-doped *h-*YMnO_3_ with and without the spin texture. While there exist a couple of slight differences when comparing the results in Fig. 4a and b over the full Brillouin zone, we found a particularly large difference in the intensity of the 5 meV mode near the C point. Complementary calculations without the spin texture confirmed that the 5 meV mode becomes strongly suppressed due to the non-magnetic impurities (Supplementary Fig. S10). In comparison, there is no noticeable suppression of the 5 meV mode in our experimental data and the theoretical calculation with the spin texture (Fig. 4c). This result implies that the in-plane spin texture formation is a key factor in retaining the 5 meV mode’s stability against the non-magnetic impurity.

To further understand the origin of the significant difference at the C point near 5 meV between Fig. 4a and b, we analyzed the eigenvector of the 5 meV magnon mode at the C point in pure *h-*YMnO_3_ (Supplementary Fig. S10). This exercise allows us to examine why it becomes particularly susceptible to the vacancy at the C point as a perturbation. As expected, an out-of-plane motion is dominant for the spin precession of the 5 meV mode, in accordance with the fact that this mode is gapped by the easy-plane anisotropy *D*_1._ However, we also found some finite in-plane precession components in its eigenvector. Notably, the in-plane precession of the six spins nearest to a specific site for the 5 meV magnon mode is almost identical to the effect of the spin canting of six spins due to the vacancy (see Supplementary Fig. S10). In other words, the 5 meV eigenmode will undergo significant energy (eigenvalue) variation due to the vacancy, indicating its sensitivity to the vacancy as a perturbation. Moreover, since the spin precession is synchronized over the triangular lattice due to a zero magnon wave-vector at the C point, the effects of this sensitivity will be amplified, leading to an ill-defined spectrum of the 5 meV mode seen in Fig. 4b. While further confirmation of whether such a minor portion of the spin precession can result in the significant change is required, these results imply that spin textures may play an important role in explaining the diluted noncollinear magnets’ magnetic excitations.

Although our INS data of YMn_1-*x*_Al_*x*_O_3_ together with the model calculations have provided some valuable insight about the spin texture, we would like to note that such an approach would be somewhat close to the indirect examination. Therefore, further measurements to directly detect the spin texture in real space will be of great help to deeper understanding of it. For instance, small angle neutron scattering (SANS) may give further hidden information about its spatial correlation, which is the key feature of the spin texture.

In summary, our work presents a unique experimental study on the energy and momentum-resolved spin dynamics of a diluted frustrated magnet. It contributes to answering the critical fundamental problem of the nontrivial impurity effects in frustrated magnets. Furthermore, our results provide the first experimental confirmation of the impurity-induced spin textures, which have been long-advocated theoretically. We demonstrate that generating a spin texture can be easily achieved using frustrated noncollinear magnets. It may also be conceivable that this giant spin texture can be manipulated and so used as potential applications as done for skyrmion.

## Methods

### Sample preparation

*h*-YMn_1−*x*_Al_*x*_O_3_ (*x* = 0, 0.02, 0.05, 0.10, 0.15, 0.20) single crystals were grown by the optical floating zone technique. Polycrystalline *h*-YMn_1−*x*_Al_*x*_O_3_ was first prepared by using Y_2_O_3_, Mn_2_O_3_ and Al_2_O_3_/Ga_2_O_3_ with a standard solid-state reaction method. The starting materials were mixed in stoichiometric ratio, and were pelletized and sintered for several times. The final sintering was done at 1300 °C for 24 hrs. 4 mm diameter feed and seed rods were prepared using the polycrystalline *h*-YMn_1−*x*_Al_*x*_O_3_ with correct compositions. Finally, *h*-YMn_1−*x*_Al_*x*_O_3_ single crystal was grown by a floating zone furnace (Crystal Systems, Japan) with the growth speed of 2 mm/h under ambient conditions. Using the IP-XRD Laue Camera (TRY-IP-YGR, IPX Co., Ltd. Japan) and the high-resolution single-crystal X-ray diffractometer (XtaLAB P200, Rigaku Japan), we confirmed the high quality of the crystal^23^ (see Supplementary Fig. S3). Further characterization was done by measuring the field-cooled and zero-field-cooled DC magnetic susceptibility and AC susceptibility (MPMS-XL5 and MPMS-3, Quantum Design USA), which confirmed the long-range order without the spin-glass signature in *h*-YMn_1-x_Al_x_O_3_. Some of the results are summarized in Fig. 1c. For inelastic neutron scattering experiments, the samples were cut into pieces with smaller sizes and were co-aligned on Al sample holders (2.2 g for *h*-YMnO_3_, 3 g for *h*-YMn_0.95_Al_0.05_O_3_ and *h*-YMn_0.9_Al_0.1_O_3_, and 1.5 g for *h*-YMn_0.85_Al_0.15_O_3_).

### Inelastic neutron scattering (INS) experiments

We carried out INS experiments on the single crystal *h*-YMn_1-*x*_Al_*x*_O_3_ using two time-of-flight (ToF) spectrometers: the MAPS spectrometer at ISIS, UK^39^ for *h*-YMnO_3_, and the 4SEASONS spectrometer at J-PARC, Japan^40^ for *h*-YMn_0.9_Al_0.1_O_3_ and *h*-YMn_0.85_Al_0.15_O_3_. In the case of *h*-YMnO_3_, the data were collected at 4 K with the incident neutron energy (*E*_*i*_*)* of 30 meV. The chopper frequency was set to 350 Hz, which yields a resolution of 0.50 *~* 0.80 meV depending on the energy transfer, as shown in Supplementary Fig. S4. For *h*-YMn_1−*x*_Al_*x*_O_3_ with *x* = 0.1 and 0.15, the data were collected at 5 K with multiple *E*_*i*_ (6.8, 10, 16, 30, and 75 meV) and the Fermi chopper frequency of 250 Hz (see Supplementary Fig. S4 for the instrumental resolution), thanks to the repetition-rate-multiplication (RRM) method implemented in 4SEASONS^41^. In all experiments, the samples were mounted in the geometry of (HHL) plane horizontal and were rotated during the measurement. For the data analysis, we used the Utsusemi^42^ and Horace software^43^. Considering the crystal and magnetic symmetry of *h*-YMnO_3_, the data were symmetrized into the irreducible Brillouin zone, which reduced the data’s statistical error. Also, as the magnon modes’ dispersion along the *c*^∗^-axis is negligible in *h*-YMn_1-*x*_Al_*x*_O_3_, the data were integrated over the *c*^∗^-axis direction in a range of *L*= [*−*3, 3].

To acquire further information, we also carried out INS experiments in the Taipan triple-axis spectrometer at ANSTO, Australia, for *h*-YMnO_3_ and *h*-YMn_0.95_Al_0.05_O_3_. The data were collected at 5 K with the scattered neutron energy of 14.86 meV.

### Magnon linewidth analysis

To estimate the **Q**-dependence of magnon energy linewidth, we performed magnon peak fittings at the **Q** points within the full Brillouin zone. We used a Lorentzian function to fit magnon peaks, while a Gaussian function fitted the incoherent quasi-elastic signal. Instrumental resolution effects were removed from the fitted HWHM, assuming that the following relation holds:

2 $${{\rm{HWHM}}}_{\rm{f i t}}=\sqrt{\,{({{\rm{HWHM}}}_{{\rm{instrument}}})}^{2}+{({{\rm{HWHM}}}_{{\rm{intrinsic}}})}^{2}},$$

where HWHM_instrument_ was derived from the profile shown in Supplementary Fig. S4. Note that for the doped samples, HWHM_intrinsic_ is much larger than HWHM_instrument_ at most **Q** points (${{\rm{HWHM}}}_{{\rm{intrinsic}}}\cong 10\,{{\rm{HWHM}}}_{{\rm{instrument}}}$), which guarantees the validity of Eq. 2. As there is more than one magnon peak at a certain **Q** point, HWHM values of the magnon peaks were averaged at each **Q** point, the results of which are displayed in Figs. 3d–f.

### Theoretical calculations

To take the dilution effect into account when performing spin-wave calculations, randomly distributed vacancies were created into a two-dimensional triangular lattice of 30 *×* 30 sizes with periodic boundary conditions. The resultant magnetic ground state affected by the vacancies was derived by simulated annealing followed by the conjugate gradient method. Using LSWT, we diagonalized the spin Hamiltonian (Eq. 1) with the ground state obtained from the previous process and calculated corresponding INS cross-sections using the SpinW library^44^. To get a statistically good result, we averaged the calculated INS cross-sections over 40 impurity replicas. We applied the same method to calculate the spin-wave spectra in a diluted square lattice antiferromagnet (see Supplementary Fig. S9) and a diluted TLAF without spin texture. Although 120˚ magnetic order does not correspond to a classical energy minimum in a diluted TLAF, the validity of such examination still holds as far as the resulting magnon spectra are not ill-defined.

For precise comparison with the data, we performed energy and momentum resolution convolution based on the technical information of each ToF beamline (see Supplementary Fig. S4). Notably, the effect of data integration over [00 *L*] was included by calculating the average of the INS cross-sections with different L values (at 0.1 r.l.u. steps). The weighting factor of each piece was determined by the histogram of detector counts included in the data plot as a function of *L*. As a result, we confirmed a good agreement between the calculated magnon spectra and the data (Supplementary Fig. S6).

## Supplementary information

Supplementary Information

## Data Availability

The data used in this study are available from the corresponding author upon request.

## Code availability

Custom codes used in this article are available from the corresponding author upon request.
