# Supplementary material for: Spin texture induced by non-magnetic doping and spin dynamics in 2D triangular lattice antiferromagnet h-Y(Mn,Al)O3
Source: Nat Commun. 2021 Apr 16;12:2306. doi: 10.1038/s41467-021-22569-3 (PMC8052335; doi:10.1038/s41467-021-22569-3)
Supplement: Supplementary file 1 — Supplementary Information [file 41467_2021_22569_MOESM1_ESM.pdf]

Supplementary Information: Spin texture induced by nonmagnetic doping and spin dynamics in 2D triangular lattice antiferromagnet *h*-Y(Mn,Al)O_3

Pyeongjae Park[1,2,3†], Kisoo Park[2,3†], Joosung Oh[2,3], Ki Hoon Lee[2,3,4,5], Jonathan C. Leiner[2,3,6], Hasung Sim[2,3], Taehun Kim[1,2,3], Jaehong Jeong[1,2,3], Kirrily C. Rule[7], Kazuya Kamazawa[8], Kazuki Iida[8], T. G. Perring[9], Hyungje Woo[9,10], S.-W. Cheong[11], M. E. Zhitomirsky[12], A. L. Chernyshev[13] & Je-Geun Park[1,2,3*]

[1]*Center for Quantum Materials, Seoul National University, Seoul 08826, Republic of Korea*
[2]*Center for Correlated Electron Systems, Institute for Basic Science, Seoul National University, Seoul 08826, Republic of Korea*
[3]*Department of Physics and Astronomy & Institute of Applied Physics, Seoul National University, Seoul 08826, Republic of Korea*
[4]*Center for Theoretical Physics of Complex Systems, Institute for Basic Science, Daejeon 34126, Korea*
[5]*Department of Physics, Incheon National University, Incheon 22012, Korea*
[6]*Physik-Department, Technische Universität München, D-85748 Garching, Germany*
[7]*Australian Nuclear Science and Technology Organisation, Lucas Heights, 2234 New South Wales, Australia*
[8]*Comprehensive Research Organization for Science and Society (CROSS), Tokai, Ibaraki 319-1106, Japan*
[9]*ISIS Pulsed Neutron and Muon Source, STFC Rutherford Appleton Laboratory, Didcot, Oxfordshire, OX11 0QX, United Kingdom*
[10]*Department of Physics, Brookhaven National Laboratory, Upton, New York 11973, USA*
[11]*Department of Physics and Astronomy, and Rutgers Center for Emergent Materials, Rutgers University, Piscataway, New Jersey 08854, USA*
[12]*Université Grenoble Alpes, CEA, IRIG, PHELIQS, 38000 Grenoble, France*
[13]*Department of Physics and Astronomy, University of California, Irvine, California 92697, USA*
* Corresponding author: jgpark10@snu.ac.kr

Supplementary Note. Spin texture in (anti-)trimerized triangular lattice antiferromagnet (TLAF)

As the spin dynamics of h-$YMnO_3$ can be well explained by the spin Hamiltonian without a trimerization effect[1], we did not consider trimerization in our model calculation. Haing said that, it is still worth investigating whether trimerzation in h-$YMnO_3$ gives noteworthy changes on the spin texture. Therefore, we examined the change of spin texture due to (anti-)trimerzation by giving exchange interactions with different sizes to trimerized bonds and anti-trimerized bonds, respectively. Based on the previous studies[2,3] on the spin dynamics of h-$YMnO_3$, we chose $J_2 = 0.8J_1$ (a trimerized case) and $J_2 = 1.2J_1$ (an anti-trimerzed case).

Using the same procedure described in the Method of the main text, we calculated the canted ground state due to a non-magnetic impurity with (anti-)trimerization (anti-)trimerized TLAF. Fig. S2 shows the canting angle of spins $|\delta\Theta(r)|$ as a function of the distance from the impurity site (r) on a logarithmic scale, both with and without (anti-)trimerzation. The result demonstrates that the aspect of spin canting in (anti-)trimerized TLAF remains similar to that in ideal TLAF at the intermediate region with little finite size effects (r<30). Therefore, our model calculations without the trimeriation effect well represents the possible spin texture and correspondsing spin dynamics in h-$YMn_xAl_{1-x}O_3$.

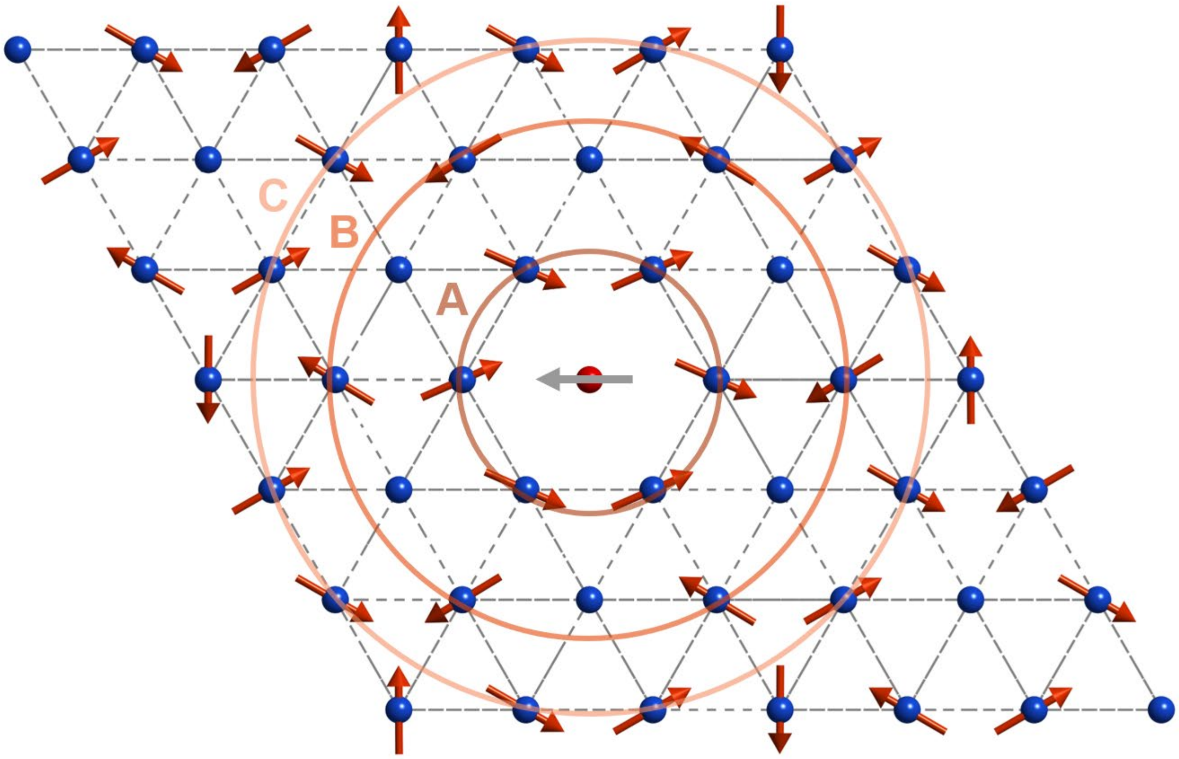

Fig.S1 | The normalized difference between the spin configuration with and without spin texture, i.e., $\vec{\mathbf{S}}_{canted} - \vec{\mathbf{S}}_{120°}$. A gray arrow at the center denotes the net magnetic moment (-S_0) introduced by a non-magnetic impurity. Spins on the same circle (A~C) has the similar canting direction (left or right), which demonstrate the spatial structure of spin texture. Sites without a red arrow mean zero spin canting. Note that magnitude of the net difference at each site is normalized to the identical value for better demonstration.

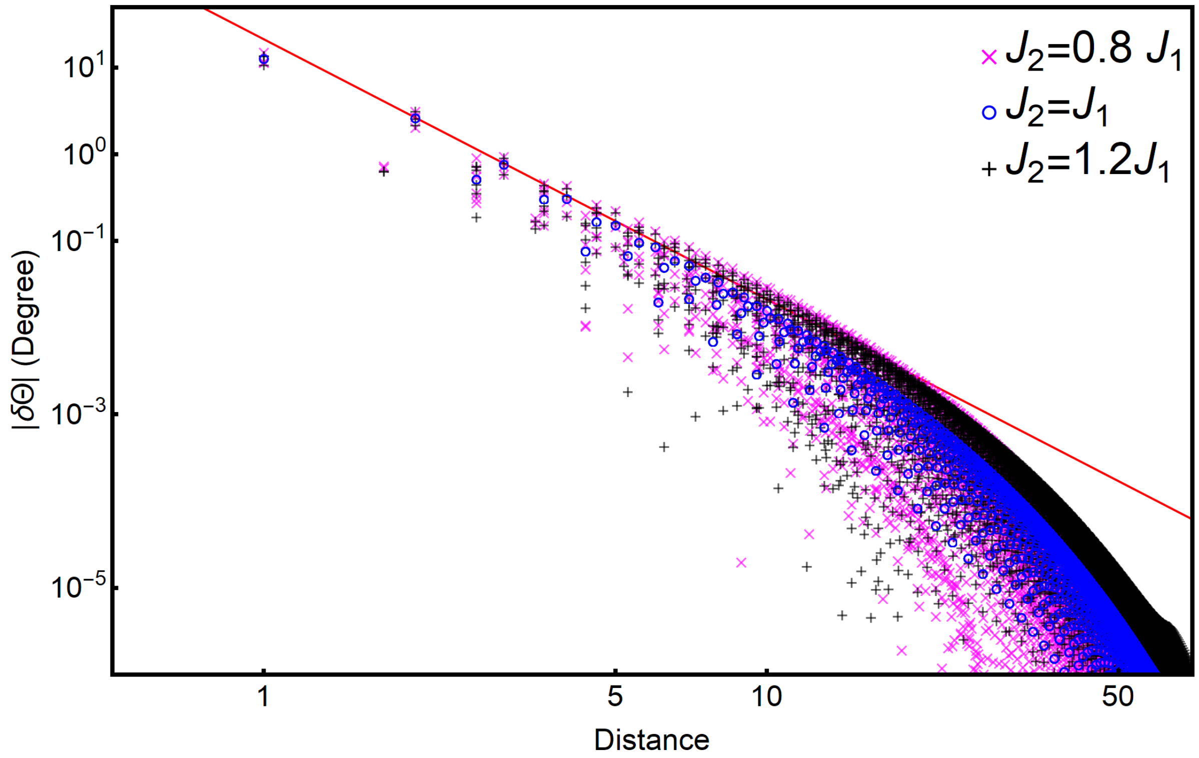

Fig.S2 | Distance dependence of the canting angle $|\delta\Theta(r)|$ in the spin texture around a vacancy for an ideal (o), a trimerized (x) , and an anti-trimerized triangular lattice antiferromagnet (+). The red line is a guided plot of the $1/r^3$ behavior. We used the notation of J_1 and J_2 same as those in refs. 1-3 of the supplementary information. The figure clearly shows that the spin texture of the (anti-)trimerized case remains similar to that of an ideal triangular lattice.

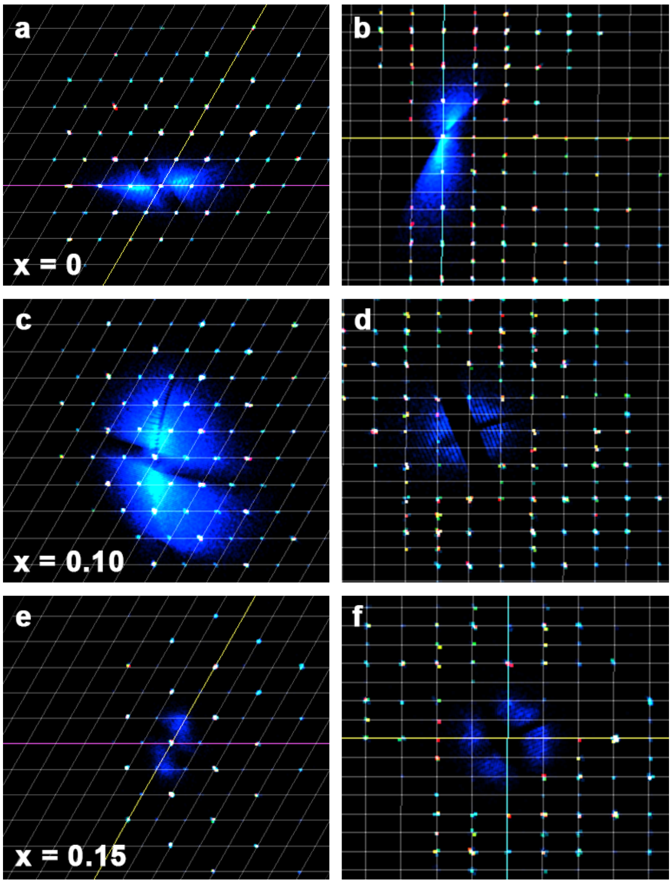

Fig.S3 | X-ray diffraction patterns of **a,b** *h*-YMnO3, **c,d** *h*-$YMn_{0.9}Al_{0.1}O_3$, and **e,f** *h*-$YMn_{0.85}Al_{0.15}O_3$ single crystals mapped on the a^*-b^* and b^*-c^* planes of the reciprocal space.

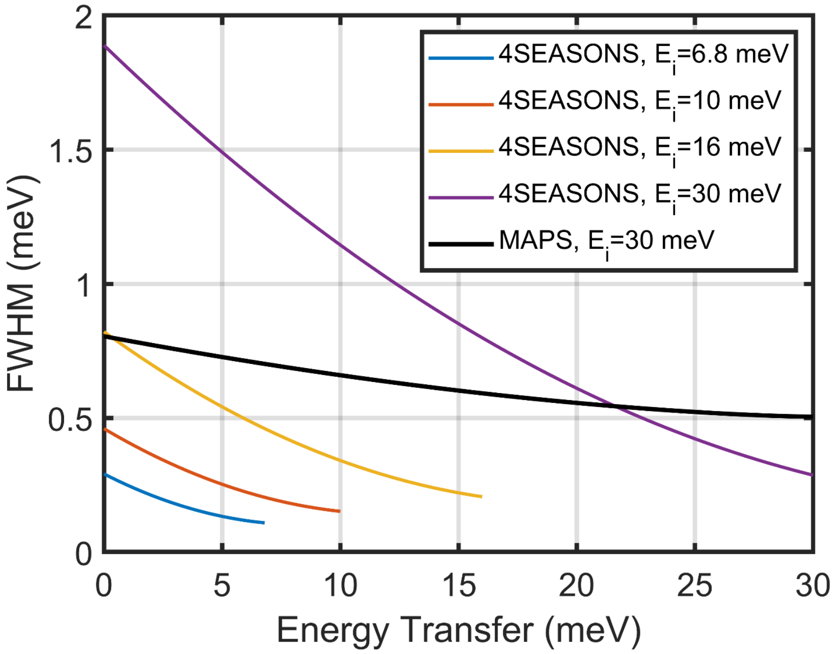

Fig.S4 | Instrumental resolution of the time-of-flight neutron spectrometers used in this study. A solid black line denotes the instrumental resolution of the MAPS beamline with E_i = 30 meV and a Fermi chopper frequency of 350 Hz. Colored lines are the instrumental resolution of the 4SEASONS beamline with E_i = 6.8, 10, 16, 30, and 75 meV and a Fermi chopper frequency of 250 Hz when using the repetition-rate multiplication method.

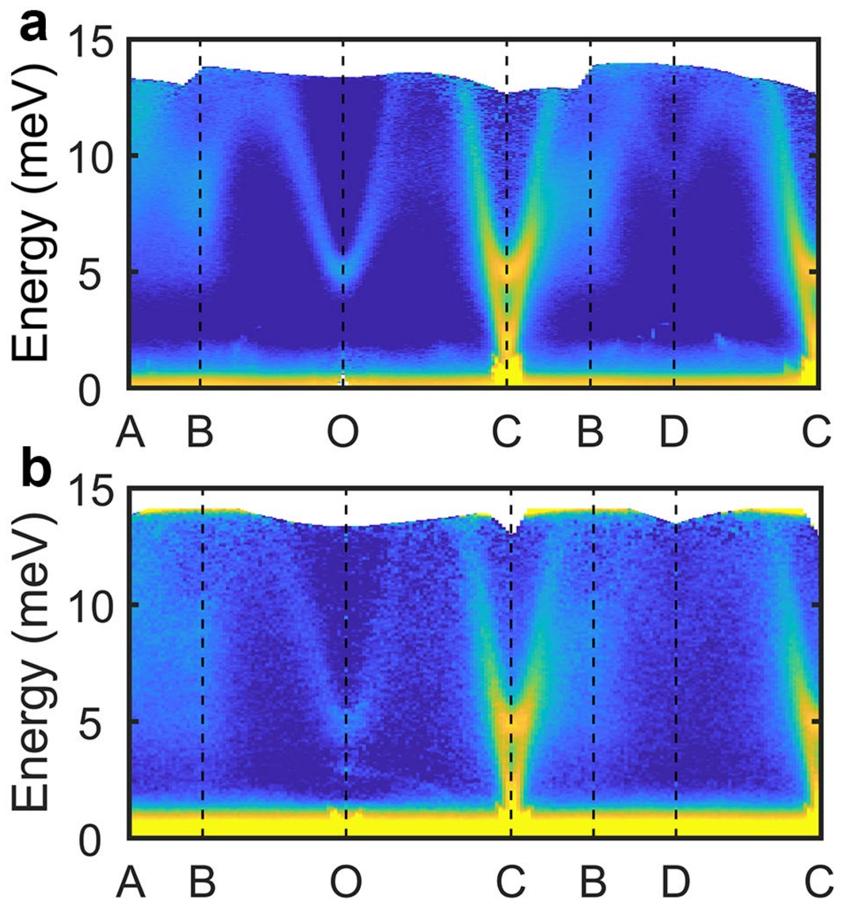

Fig.S5 | INS spectra of **a** h-$YMn_{0.9}Al_{0.1}O_3$ (J-PARC), and **b** h-$YMn_{0.85}Al_{0.15}O_3$ (J-PARC) measured at 5 K with the incident neutron energy of E_i = 16 meV. The scattering intensity of the data was integrated over the c^*-axis. Having better instrumental resolution than the data in Fig. 2, these data clearly show the severe broadening of the magnon modes at the zone boundary due to the doping, which is in stark contrast to the robustness of the magnon modes near the zone center (C point).

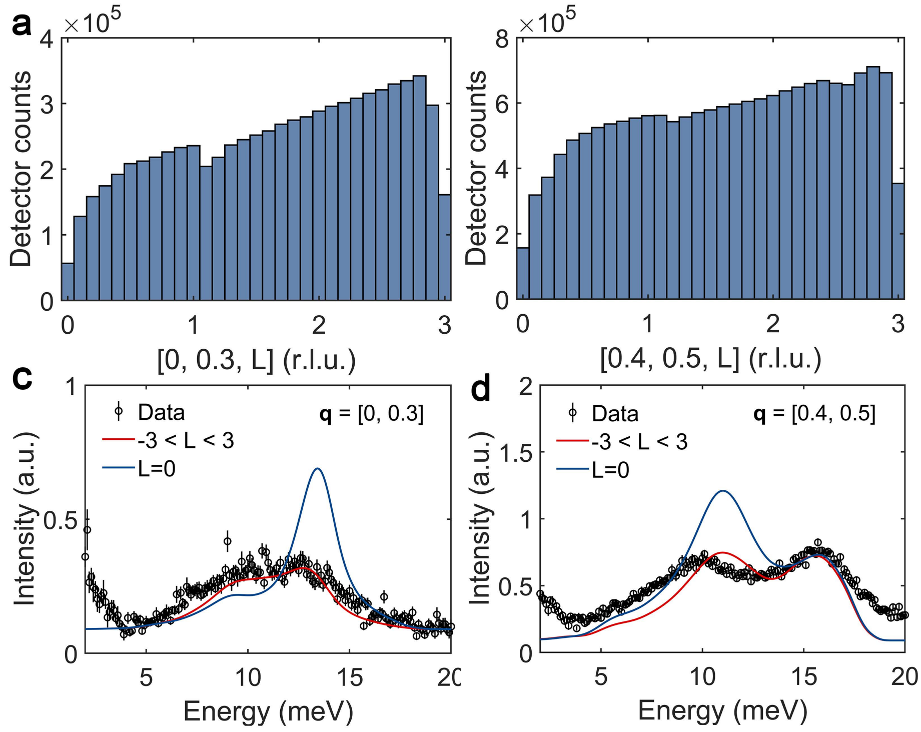

Fig.S6 | Data integration effects along the *c**-axis for *h*-$YMn_{0.9}Al_{0.1}O_3$**. a-b,** Histograms showing the individual momentum transfer of the detector counts included in the const-**Q** cuts at **a** [0, 0.3] and **b** [0.4, 0.5] (r.l.u.). They were used to consider the intensity integration along the *c**-axis in the calculation. **c-d,** Const-**Q** cuts at [0, 0.3] and [0.4, 0.5] (r.l.u.), and the calculated spin-wave spectra with (solid red lines) and without (solid blue lines) the data integration effect along the *c**-axis.

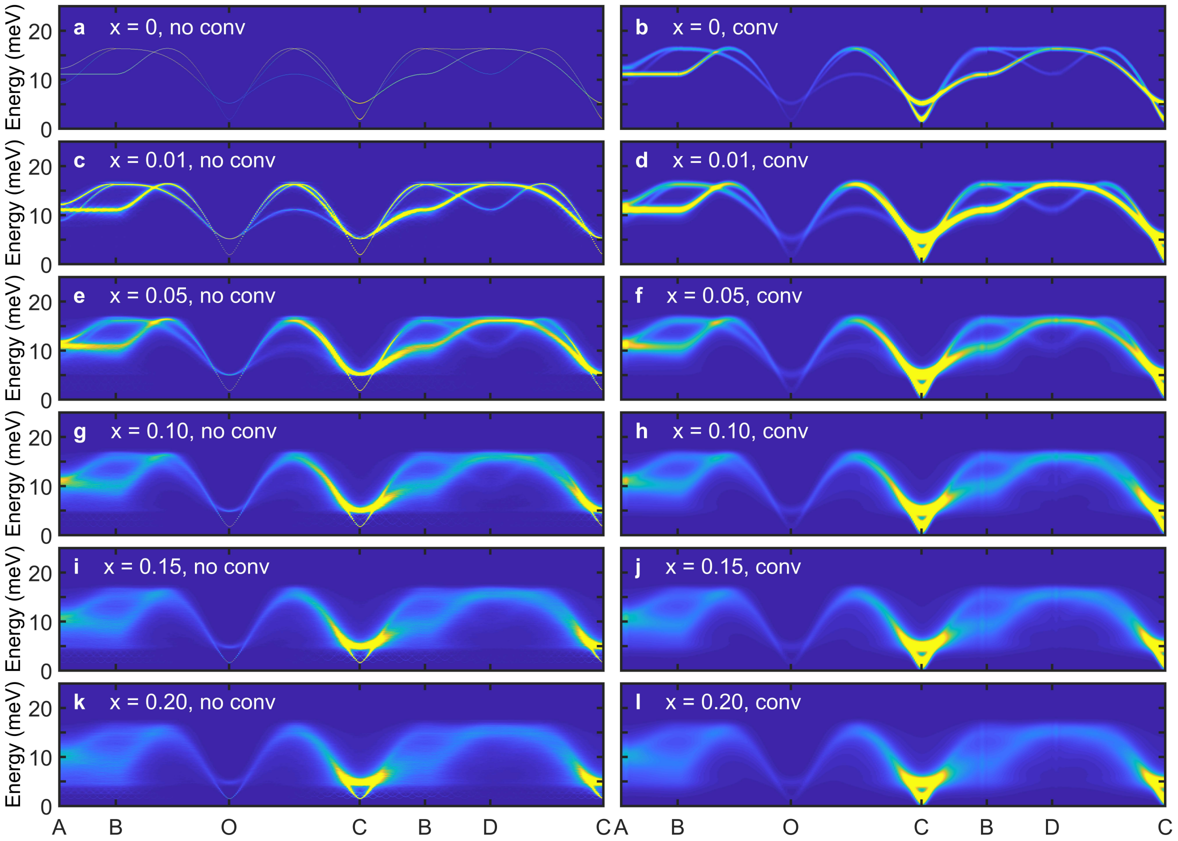

Fig.S7 | Theoretically calculated INS cross-sections of *h*-$YMn_{1-x}Al_xO_3$ with (a right panel) and without (a left panel) the instrumental resolution convolution. The color plots without the resolution convolution clearly show the intrinsic linewidth of magnon modes as well as the downward shift of the magnon dispersion along the A-B line. Note that the calculations in this figure do not include the data integration effect along the *c**-axis.

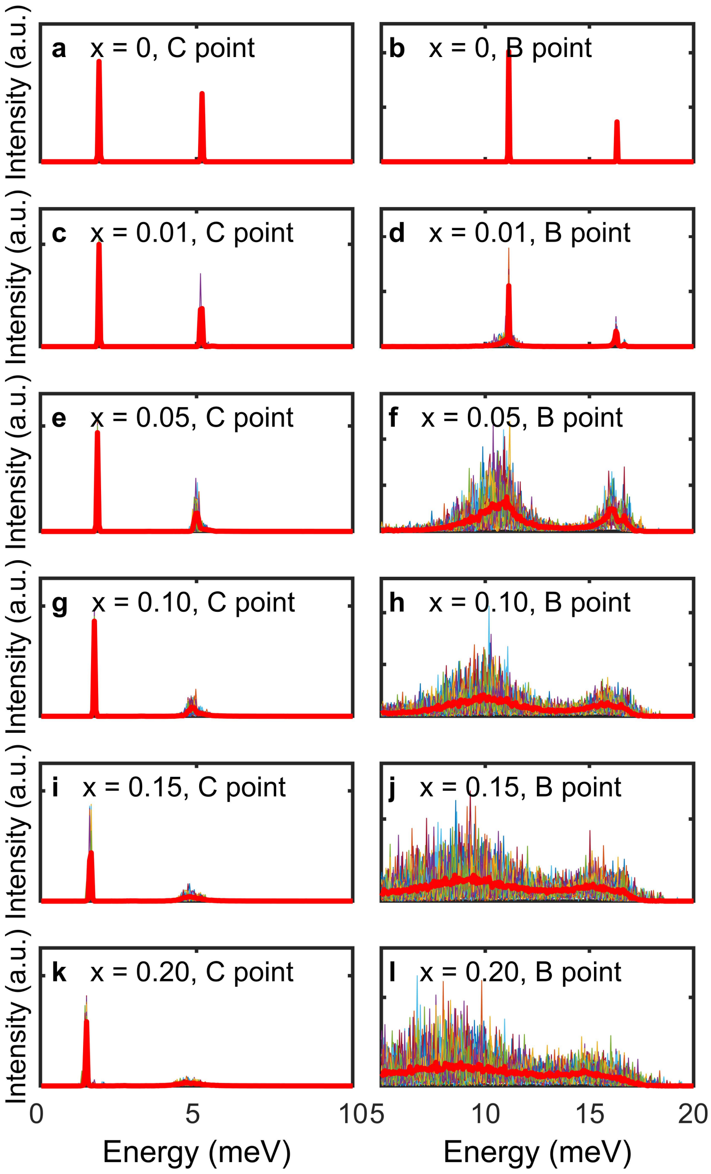

Fig.S8 | a-c, Constant-**Q** cuts of the theoretically calculated INS cross-sections for *h*-$YMn_{1-x}Al_xO_3$ at the C point (left) and the B point (right). Thin lines are the calculated INS cross-sections from 40 replicas with random impurities (see Methods), which were used to calculate the ensemble average of the INS cross-sections. Red thick lines are the ensemble-averaged INS cross-sections of the 40 replicas. Comparing the results at the C point and the B point clearly show that the broadening effect under the doping is strongly **Q**-dependent. Note that the calculations in this figure do not include the data integration effect along the *c**-axis.

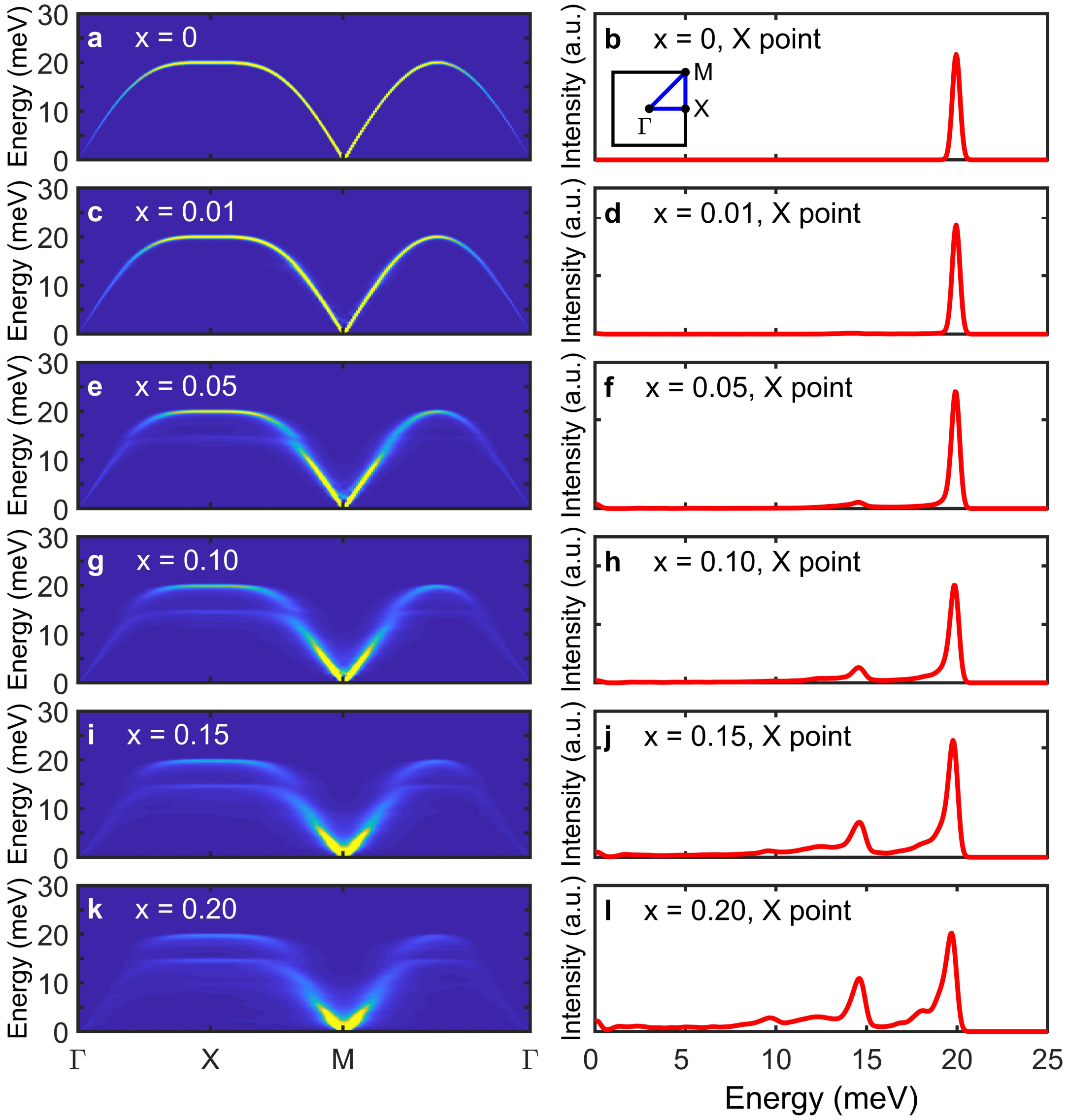

Fig.S9 | (Left) Calculated dynamical structure factor along the high symmetric lines in a diluted square lattice antiferromagnet with different amounts of doping. (Right) Constant **Q**-cuts of the results in the left panels at the X point.

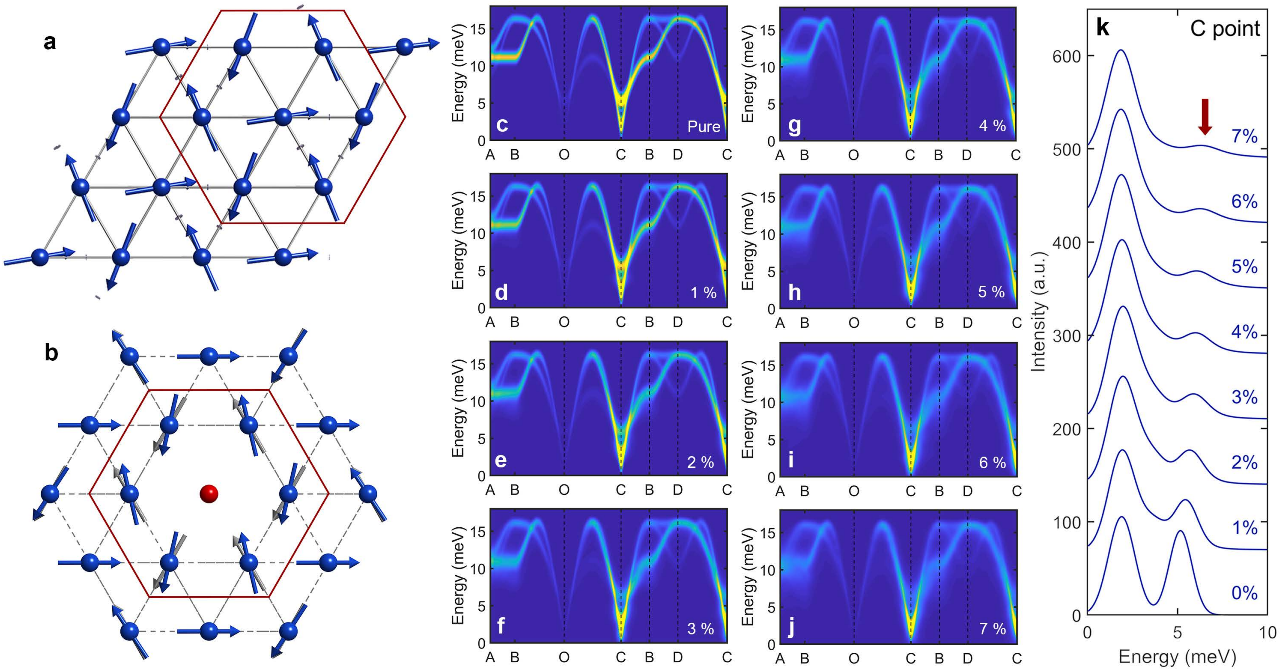

Fig.S10 | Selective vulnerability of the 5 meV magnon mode to the nonmagnetic impurity. **a,** A snapshot of the real-space spin precession for the 5 meV magnon mode at the C point. **b,** Spin canting due to the partial relief of the geometrical frustration by a nonmagnetic impurity (the same figure as Fig. 1a in the main text). Comparison between **a** and **b** shows the coincidence between the spin precession of the 5 meV mode and the spin canting due to the impurity. **c-j,** Doping dependence of the spin-wave spectra without considering the spin texture. **k,** Constant-**Q** cuts of the calculation results in **c-j** at the C point, which demonstrates that the 5 meV mode (the red arrow) undergoes a significant change under the doping. Note that the calculations in this figure do not include the data integration effect along the c^*-axis.

References

1 Oh, J. *et al.* Spontaneous decays of magneto-elastic excitations in non-collinear antiferromagnet (Y,Lu)MnO3. *Nature Communications* **7**, 13146 (2016).

2 Oh, J. *et al.* Magnon Breakdown in a Two Dimensional Triangular Lattice Heisenberg Antiferromagnet of Multiferroic $LuMnO_3$. *Physical Review Letters* **111**, 257202 (2013).

3 Sato, T. J. *et al.* Unconventional spin fluctuations in the hexagonal antiferromagnet $YMnO_3$. *Physical Review B* **68**, 014432 (2003).
